# The Role of Secondary Cytoreduction and Hyperthermic Intraperitoneal Chemotherapy in Recurrent Ovarian Peritoneal Carcinomatosis

**DOI:** 10.3390/jpm14070742

**Published:** 2024-07-11

**Authors:** Pirilti Özcan, Özgül Düzgün

**Affiliations:** 1 Department of General Surgery, Cerrahpaşa Faculty of Medicine, Istanbul University, 34116 Istanbul, Turkey; 2 Department of Surgical Oncology, İstanbul Umraniye Training and Research Hospital, University of Health Sciences, 34764 Istanbul, Turkey; ozgul.duzgun@sbu.edu.tr

**Keywords:** ovarian cancer, recurrent, secondary cytoreductive surgery, peritoneal carcinomatosis

## Abstract

Background and Aims: Ovarian cancer maintains the highest mortality rate among gynecological malignancies. Unfortunately, two-thirds of cases are diagnosed at an advanced stage with the presence of peritoneal carcinomatosis. In this study, we aimed to present the 7-year results of cytoreductive surgery and hyperthermic intraperitoneal chemotherapy in cases where peritoneal carcinomatosis developed during the medical oncological treatment and follow-up after primary high-grade serous ovarian cancer debulking surgeries. Patients and Methods: Data from 63 patients collected prospectively in our clinic were retrospectively evaluated. Results: Postoperative Clavien–Dindo grade 3–4 complications occurred in 12 cases (19%) and 14 cases (22.2%), respectively. CD grade 3a complications developed in four cases (6.3%), which were treated with percutaneous drainage catheters, while CD grade 3b complications occurred in eight cases (12.7%), and these cases underwent reoperation. Five cases (7.9%) experienced mortality within the first 30 days. The mean survival time was determined as 44.99 months (36.33–53.65), while the median survival time was 56 months. Conclusions: In selected patients requiring redo surgery due to recurrent ovarian cancer, secondary cytoreductive surgery and hyperthermic intraperitoneal chemotherapy are associated with longer overall survival and should be considered in the treatment of advanced-stage disease. Further large-scale randomized controlled trials are needed in this regard.

## 1. Introduction

Ovarian cancer (OC) maintains the highest mortality rate among gynecological malignancies. Unfortunately, two-thirds of cases are diagnosed at an advanced stage with the presence of peritoneal carcinomatosis (PC). It is known that the natural course of OC, especially from FIGO stage III onwards, is characterized by peritoneal recurrences in 70% of cases [1,2,3]. In the past 30 years, the effectiveness of cytoreductive surgery and hyperthermic intraperitoneal chemotherapy (CRS + HIPEC) has been proven in PC cases related to appendiceal pseudomyxoma and epithelioid-type mesothelioma, and it has now been included in guidelines and started to be used in PC associated with high-grade serous ovarian cancer (HGSOC) [4].

Following effective CRS, HIPEC enhances drug distribution on the peritoneal surface with the advantage of minimal systemic side effects, and it can improve outcomes by more efficiently eliminating residual microscopic peritoneal disease compared to intravenous chemotherapy [5]. Particularly in HGSOC, despite the increasing interest in CRS+HIPEC and the experimental nature of HIPEC in most national and international guidelines, it must be acknowledged that there is a controversial approach to PC associated with HGSOC due to significant differences in implementation. Spiliotis et al. and van Driel et al. also reported the results of a phase 3 randomized controlled study and the benefits of CRS following neoadjuvant chemotherapy. Among women with HGSOC, CRS+HIPEC has been shown to result in longer survival compared to CRS alone [3,4,5,6,7,8,9]. Therefore, HIPEC may be one of the new therapeutic strategies for such disseminated peritoneal lesions. In their meta-analysis study, Corte et al. investigated the effect of CRS and HIPEC after administering neoadjuvant chemotherapy in advanced epithelial ovarian cancers. They found that it has a positive effect on median overall survival (OS) and median progression-free survival (PFS) [10].

Another problematic area for high-grade serous ovarian cancer is the issue of recurrence in stage 3–4 cases despite cytoreduction, platinum compounds, anti-angiogenic agents, and polymerase inhibitors. Both the development of platinum resistance and clinical symptoms due to intra-abdominal organ metastases continue to pose challenges. Secondary SCS+HIPEC (SCS+HIPEC) in recurrent OC (ROC) has been shown to be successful without increasing operative morbidity and mortality [8,9].

In this study, we aimed to present the 7-year results of SCS+HIPEC in cases of primary HGSOC where PC developed during medical oncological treatment and follow-up after HGSOC surgery.

## 2. Patients and Methods

### 2.1. Patient Information and Ethical Considerations

Between May 2016 and 2023, data from 63 patients who underwent SCS+HIPEC due to PC during medical oncological treatment and follow-up in external clinics related to HGSOC were prospectively collected and retrospectively evaluated at the Surgical Oncology Clinic of Istanbul University Health Sciences University Ümraniye Training and Research Hospital. Informed consent forms stating that data from all cases would be used were obtained during their hospitalization. Ethical approval was obtained from the hospital ethics committee under the reference number 2023/212. All cases were evaluated by a multidisciplinary tumor board during the preoperative period. The patients’ demographic data, ASA (American Society of Anesthesiologists) scores, ECOG (Eastern Cooperative Oncology Group) scores, Body Surface Area (BSA), previous surgical operations, chemotherapy protocols, Peritoneal Carcinomatosis Index (PCI), Completeness of Cytoreduction (CC) score, and duration of intensive care unit (ICU) and hospital stay were evaluated in terms of postoperative morbidity–mortality and overall survival (OS).

### 2.2. Details of SCS and HIPEC

During the preoperative period, MRI was used for abdominal imaging and PET-CT was used to investigate distant metastases in all cases. Cases with a progressive increase in CA-125 values (average CA 125 value: 350 (100–2220)) during medical oncological treatment and follow-up and cases resistant to systemic treatment were evaluated radiologically by the multidisciplinary tumor council. Diagnostic laparoscopy was performed for all patients. Patients with a PCI score of 18 or higher were not operated on and were referred to medical oncology. Patients who were deemed operable underwent a standard incision from the xyphoid to the pubis. The PCI score was calculated. All patients had previously undergone optimal debulking surgery or surgical staging for ovarian cancer at an external center, which included some or all of the following procedures: Total Abdominal Hysterectomy + Bilateral Salpingo-Oophorectomy + Pelvic Para-Aortic Lymph Node Dissection (TAH+BSO+PPALND) + Total Omentectomy. For these patients who had undergone previous surgery, peritonectomy and necessary additional organ resections were performed. Anastomoses were created before the HIPEC procedure. After the creation of stomas, the HIPEC procedure was initiated. After closure of the abdominal skin, intraperitoneal chemotherapy consisting of cisplatin and doxorubicin was administered for 60 min. No major complications related to chemotherapy were observed. HIPEC was performed using the Belmont Hyperthermia Pump (Belmont Instrument Corporation, Billerica, MA, USA) at a temperature of 43 degrees Celsius, with a flow rate of 1200 cc/h. Cisplatin (75 mg/m^2^/BSA) and doxorubicin (15 mg/m^2^/BSA) were intraperitoneally injected in a 0.9% NaCl solution for 60 min. Patients were admitted to the postoperative intensive care unit. After discharge, patients were followed up every 3 months for the first 2 years and every 6 months after the third year. Follow-up visits included tumor marker assessments, and MRI and PET-CT scans.

### 2.3. Statistical Analyses

Data were analyzed using the Statistical Package for Social Sciences version 25.0 (IBM SPSS, Chicago, IL, USA). OS was determined as the time period between the operation date and the death, and PFS as the time period between the operation date and the laboratory disease progression, while DFS was the time between the operation date and the first clinical recurrence of the disease. In the calculation of OS, PFS, and DFS, the Kaplan–Meier method was followed by the long-rank test to compare them. A Cox regression analysis was used to determine the factors that may affect survival. Quantitative variables were shown as the mean ± standard deviation (SD) being statistically significant.

## 3. Results

A total of 63 patients who had previously undergone TAH + BSO + PPALND + Total Omentectomy at external centers for ovarian PC and experienced progression while under treatment in the postoperative medical oncology unit were referred to our clinic for SCS+HIPEC. SCS+HIPEC operations were performed for these patients. All of these patients were operated on previously due to FIGO 3A-C ovarian cancer. The median platinum-free interval was 34 months and relapse free survival was 22 months. 

The average age of the patients was 58 (range: 36–77). According to the ASA score, 17 patients (27%) were classified as ASA 1, 15 patients (24%) as ASA 2, 30 patients (47.5%) as ASA 3, and 1 patient (1.5%) as ASA 4. The average BSA was 171 (range: 141–202). The ECOG score ranged from 0 to 3, with an average score of 1.2. The average PCI score was 13 (range: 3–18), and as for the CC score, CC 0 was achieved in 46 patients (71.1%), CC 1 in 7 patients (11.1%), and CC 2 in 10 patients (15.8%). While HIPEC was applied to CC 0/1 cases, HIPEC was not applied to CC 2 cases. The average duration of surgery was 7 h (range: 4–13.5). The average length of stay in the ICU was 1.6 days (range: 0-10), and the average hospital stay was 8 days (range: 4–42). A total of 15 patients (23.8%) underwent stoma creation, with 10 patients (15.9%) having a loop ileostomy and 5 patients (7.9%) having an end ileostomy (Table 1).

In addition to peritonectomies performed due to visceral and parietal involvement, partial diaphragm resection was performed in eight patients (12.7%) with full-thickness diaphragmatic involvement. In two cases where primary diaphragm closure was not feasible, closure was achieved using polypropylene mesh. Closed thoracic tube drainage was applied to all of these cases. Liver metastasectomies were performed in 17 patients (26.9%) with liver metastases. Total, subtotal, and wedge resections were performed in 12 patients (19%) with gastric involvement (1 case each for total and subtotal resections, and 10 cases for wedge resections, accounting for 1.5% each). A total of 13 patients (20.6%) underwent distal pancreatectomy. Total splenectomy was performed in 17 patients (26.9%), cholecystectomy in 22 patients (34.9%), colectomy in 34 patients (53.9%), rectum resection in 29 patients (46%), partial small bowel resection in 30 patients (47.6%), partial bladder resection in 13 patients (20.6%), right nephrectomy in 1 patient (1.5%), right ureter resection + right nephrostomy in 1 patient (1.5%), partial ureter resection + ureteroneocystostomy in 3 patients (4.7%), ileoanal J-pouch anastomosis in 1 patient (1.5%), and ileorectal J-pouch anastomosis in 2 patients (3.1%) (Table 2).

In the postoperative period, the following complications were observed: In one patient (1.5%), there was bile leakage (CD 3a) due to liver metastasectomy, which was treated with a percutaneous drainage catheter. Two patients (3.1%) experienced bladder atony (CD 3a), which was managed by following up with a Foley catheter until recovery. One patient (1.5%) developed sigmoid colon perforation (CD 3b) and underwent resection, anastomosis, and loop ileostomy. One patient (1.5%) experienced hypoglycemic shock (CD 4b) secondary to medical treatment and was admitted to the ICU. Two patients (3.1%) developed ileus (CD 3b) and underwent bridectomy through surgery. Two patients (3.1%) had small bowel perforation (CD 3b) and underwent loop ileostomy. One patient (1.5%) underwent ploreken application in interventional radiology due to pleural effusion (CD 3a). In two patients (3.1%), evisceration (CD 3b) occurred, and wound closure was performed in the operating room. One patient (1.5%) experienced postoperative massive hemorrhage (CD 3b) and underwent a packing procedure. In one patient (1.5%), on the third day after surgery, urine leakage was detected from the abdominal drain, indicating left ureter injury (CD 3a), and a left nephrostomy procedure was performed in interventional radiology (Table 3).

Postoperative morbidity according to the Clavien–Dindo (CD) grading system is as follows: CD grade 1–2 complications occurred in 12 patients (19%).CD grade 3–4 complications occurred in 14 patients (22.2%). CD grade 3a complications occurred in four patients (6.3%), and they were treated with percutaneous drainage catheters. CD grade 3b complications occurred in eight patients (12.7%), and these patients required a repeat surgery. CD grade 4b complications occurred in one patient (1.5%).

**Figure 1:**
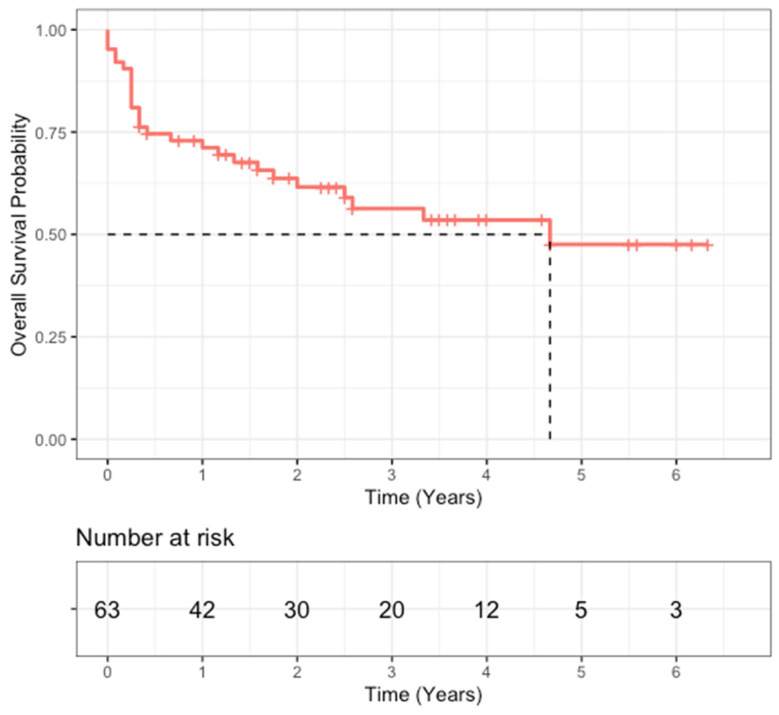
Overall Survival.

Five patients (7.9%) experienced mortality within the first 30 days. The causes of mortality included intra-abdominal bleeding requiring packing, ICU admission due to hypoglycemic shock, and intraoperative instability leading to surgery termination at CC 2 despite vasopressor treatment. The mean survival time was calculated as 44.99 months (range: 36.33–53.65), and the median survival time was determined to be 56 months (Figure 1, Table 4 and Table 5).

## 4. Discussion

In patients with advanced HGSOC, the standard treatment is primary debulking surgery followed by first-line chemotherapy [11]. Van Driel et al. in their multicenter phase 3 study in 2018 found that in stage 3 epithelial ovarian cancer, the median OS was 33.9 months in the surgery group and 45.7 months in the surgery-plus-HIPEC group. They reported that surgery-plus-HIPEC resulted in longer recurrence-free survival (RFS) and OS compared to SCS alone [10]. Prior to this study, SCS-HIPEC was considered an alternative treatment with skepticism in ovarian cancer, but after this study, the rate of SCS-HIPEC has increased in the literature and clinical practice [12,13,14].

Secondary SCS due to recurrence remains as a challenging subject. During follow-ups after initial debulking surgery, recurrences can be detected by MRI, PET-CT, or elevated CA 125 levels. However, the initial debulking surgery of these cases can differ significantly due to different surgical techniques in different institutions, different organ removals, etc., leading to a nonhomogenous distribution of these cases planned for redo surgery due to recurrence. In these patients, even if the patients have the same PCI scores, the affected organ systems are not the same. Factors such as intra-abdominal acidity, adhesions, ASA and ECOG scores of the patients, nutrition, psychiatric conditions, and the volume and experience of the centers performing the procedure further complicate these challenging secondary surgeries.

Although the goal is CC 0 resection, sometimes it is challenging to achieve CC 0/1 resection in some patients. Harter et al. and Acs et al. determined the rates of complete cytoreduction due to recurrent ovarian cancer as 76% and 74%, respectively [14,15]. Zivanovic et al. [16] reported a complete gross resection rate of 82%, while Fagotti et al. achieved a CC-0 rate of 95.3% in their cases of recurrent ovarian cancer (ROC) [17]. We know that the higher the CC 0 rate, the higher the survival rate. In our center, we determined a CC 0 rate of 71.1% and a CC 1 rate of 11.1% for complete cytoreduction, and we found that this rate is consistent with the literature.

Another important issue in these major surgeries is morbidity. With increasing surgical experience over the past 20 years, morbidity has been reduced from around 50% to 20%. Fagotti et al. reported a rate of 34.8% for CD 3 and above major morbidity in their series and performed reoperation in 14% of the cases, while no mortality was observed in the first 30 days [17]. In the series of Acs et al., a major morbidity rate of 20.5% was observed, with 13.6% of the cases undergoing reoperation, and 1.1% resulting in mortality [13]. Zivanovic et al. reported a major complication rate of 24% but did not report any mortality in their cases [16].

Van Driel et al. compared SCS with SCS+HIPEC in their own series and found that the incidence of grade 3 or 4 adverse events was similar in the two groups (25% in the surgery group and 27% in the surgery-plus-HIPEC group, *p* = 0.76). They reported a bowel resection rate of 24.5% and did not report the mortality rate in the SCS+HIPEC group [9]. In our series, we observed a 20.6% rate of major complications and a 12.7% rate of reoperation; 7.9% of our cases experienced mortality within the first 30 days. While our morbidity and reoperation rates are consistent with the literature, we attributed the high mortality rate to the fact that our clinical facility is newly established and we have performed a higher number of colorectal resections, splenectomies, and liver metastasectomies. Furthermore, recent studies have started to investigate the unfavorable prognostic factor of splenectomy [17,18]. Additionally, we consider it a flaw in our patient management that we did not consistently work with the same anesthesia team due to rotations in the preoperative, perioperative, and postoperative periods.

Another important issue is the lack of consensus on chemotherapy protocols, drug doses, procedure duration, and parameters of temperature and circulation in the HIPEC machine, which each clinic still determines based on its own experience. Platinum agents form the mainstay of treatment in ROC [19]. Chen et al. used a 90 min HIPEC regimen with cisplatin at a dose of 75 or 90 mg/m^2^/BSA and paclitaxel at a dose of 135 or 175 mg/m^2^/BSA, with cisplatin + paclitaxel preferred in 75% of cases, cisplatin + doxorubicin in 3.8% of cases, and mitomycin C in 11% of cases [18]. Acs et al. increased the HIPEC duration from 60 to 90 min and performed 60 min HIPEC in 43.2% of cases and 90 min HIPEC in 56.8% of cases, using cisplatin in 53.4% and cisplatin + doxorubicin in 39.8% of cases. They used cisplatin at a dose of 50–75 mg/m^2^/BSA, doxorubicin at a dose of 15 mg/m^2^/BSA, gemcitabine at a dose of 1000 mg/m^2^/BSA, and mitomycin at a dose of 30 mg/m^2^/BSA as preparations [14]. Van Driel et al. used cisplatin at a dose of 100 mg/m^2^/BSA at 40 °C during the 90 min HIPEC procedure [9]. As can be seen, there is no consensus among clinics, which is one of the weaknesses of HIPEC. In our clinic, we applied a 90 min HIPEC duration and treated all our cases with cisplatin at a dose of 75 mg/m^2^/BSA and doxorubicin at a dose of 15 mg/m^2^/BSA.

In their phase II study on secondary cytoreduction in patients with ROC and carboplatin-based HIPEC, Zivanovic et al. reported that carboplatin was well tolerated but did not demonstrate superior clinical outcomes. They concluded that HIPEC with carboplatin did not have a significant impact [16]. However, in a bicentric retrospective study by Acs et al. on the treatment of patients with platinum-sensitive ovarian cancer and SCS and HIPEC, they emphasized the effectiveness and feasibility of achieving complete cytoreduction through multidisciplinary participation, even in cases with extensive tumor involvement [14].

The PCI score continues to be the most important parameter for cytoreduction. Patients with a lower PCI score allow for less extensive resection. In Zivanovic et al.’s study [16], a 37% rate of bowel resection was reported, but information regarding other additional organ resections was not provided. Acs et al. reported 22% diaphragm resection, 40% colon resection, 28% small bowel resection, 33% rectum resection, 30% cholecystectomy, 12% liver resection, and 6.8% stomach resection in their cytoreduction procedures [14]. Fagotti et al. reported 34.9% diaphragm stripping and resection, 7% cholecystectomy, 4.6% liver resection, 30% splenectomy, and 46.5% bowel resection [17]. In our center, we performed 12.7% diaphragm, 53.9% colon, 47.6% small bowel, 46% rectum, 34.9% cholecystectomy, 27% liver, and 19% stomach resections. We observed higher rates of colorectal and liver resections in our series, which we attribute to our expertise in surgical oncology and our focus on hepatobiliary and lower gastrointestinal areas. We recognize that the success of cytoreduction is crucial for achieving positive outcomes. We understand that the effectiveness of the procedure is compromised if complete cytoreduction is not achieved, as evidenced by clinical outcomes in peritoneal carcinomatosis originating from other intra-abdominal organs. Therefore, we acknowledge the importance of aggressive surgery based on achieving an R0 resection.

In the literature, the median OS for advanced ovarian cancer has been reported to range from 28.5 to 77.8 months [20,21,22]. Filis et al. in their meta-analysis of 218 cases concluded that the use of HIPEC in ROC did not provide a survival advantage. They also found that the risk of grade ≥ 3 adverse events was similar between the HIPEC and non-HIPEC groups [12]. Chiva et al. in their recurrent cohort study reported an OS of 36.5 months (range: 23–62) after HIPEC, with a median disease-free survival of 20.2 months (range: 11–29). The rates of severe morbidity were 19% in the recurrent groups, indicating no apparent advantage of this treatment in terms of survival outcomes [23]. Chen et al. observed a median progression-free survival of 11.8 months and OS of 34.5 months in a retrospective study of 51 cases of ROC, while Zivanovic et al. reported an OS of 52.5 months [16,17,18]. Acs et al. determined a median OS of 43.1 months and a 5-year survival rate of 39.7 [14]. Van Driel et al., when comparing the surgery-only group with the surgery-plus-HIPEC group, found a median RFS of 10.7 months in the surgery group and 14.2 months in the surgery-plus-HIPEC group. The median OS was 33.9 months in the surgery group and 45.7 months in the surgery-plus-HIPEC group [9]. In our own study, we observed a mean OS of 44.99 months and a median OS of 56 months. We found that the median OS in our study was similar to that reported by Zivanovic and van Driel et al. The limitations of our study include its retrospective nature and the lack of a control group. Also, the BRCA gene status is another factor regarding limitations of this study. The presence of somatic + germline BRCA mutations in our study was 15%. A somatic mutation analysis was conducted in patients without germline BRCA mutations. A limitation of our study is that we expect better responses to oncologic treatments in BRCA mutation-positive cases compared to those without mutations due to their sensitivity to platinum chemotherapy and targeted therapies. Additionally, another limitation is the inability to assess the HRD (homologous recombination deficiency) status at our center.

## 5. Conclusions

In selected patients requiring redo surgery due to recurrent ovarian cancer, SCS+HIPEC is associated with longer OS with acceptable morbidity and mortality and this should be considered in the treatment of advanced-stage disease. There is a need for larger-scale randomized controlled studies in this regard.

## Figures and Tables

**Table 1:** Baseline Characteristics.

Variables	Total (n = 63)
Age (year)	58 (36–77)
ASA	
ASA 1 (%)	17 (26.9%)
ASA 2 (%)	15 (23.8%)
ASA 3 (%)	30 (47.6%)
ASA 4 (%)	1 (1.5%)
BSA (m^2^)	174 (141–202)
Operation time (hour)	7.2 (4–13.5)
ICU stay (day)	1.6 (0–10)
Hospital stay (day)	8 (4–42)
PCI score	13 (3–18)
CC score	
CC0 (%)	46 (73.1%)
CC1 (%)	7 (11.1%)
CC2 (%)	10 (15.8%)

BSA: Body Surface Area; ICU: Intensive Care Unit; PCI: Peritoneal Cancer Index; CC: Complete Cytoreduction. Data are presented as median (min–max) or n (%).

**Table 2:** Surgical characteristics.

Variables	Total (n = 63)
Stoma	15 (23.8%)
End ileostomy (%)	5 (7.9%)
Loop ileostomy (%)	10 (15.8%)
Organ metastasis	
Diaphragm	8 (12.7%)
Mesh reconstruction	2 (3.1%)
Liver metastasectomy	17 (26.9%)
Gastric	12 (19.0%)
Total gastrectomy	1 (1.5%)
Subtotal gastrectomy	1 (1.5%)
Wedge resection	10(15.8%)
Pancreas	13 (20.6%)
Splenectomy	17 (26.9%)
Cholecystectomy	22 (34.9%)
Colectomy	34 (53.9%)
Low anterior resection	29 (46.0%)
Partial bowel resection	30 (47.6%)
Ileoanal anastomosis with J-pouch	1 (1.5%)
Ileorectal anastomosis with J-pouch	2 (3.1%)
Nephrectomy	1 (1.5%)
Ureteroneocystostomy	3 (4.7%)
Nephrostomy	1 (1.5%)
Bladder resection	13 (20.6%)

Data are presented as n (%).

**Table 3:** Complications.

Complication	Intervention	n (%)
Ileus	Adhesiolysis	2 (3.1%)
Sigmoid perforation	Re-anastomosis + loop ileostomy	1 (1.5%)
Intestinal bowel perforation	End ileostomy	2 (3.1%)
Bleeding	Packing and depacking	1 (1.5%)
Evisceration	Abdominal closure	2 (3.1%)
Bile leakage from liver	Percutaneous drainage	1 (1.5%)
Bladder atony	Urinary catheter	2 (3.1%)
Pleural effusion	Percutaneous drainage	1 (1.5%)
Hypoglycemic shock	Pharmacological treatment	1 (1.5%)
Bladder perforation	Nephrostomy	1 (1.5%)
Total		14 (22.2%)

**Table 4:** Morbidity and Mortality for Clavien–Dindo Classification.

CD I	7 (11.1%)
CD II	5 (7.9%)
CD IIIa	4 (6.3%)
CD IIIb	8 (12.7%)
CD IVa	1 (1.5%)
CD IVb	0
CD V	5 (7.9%)

**Table 5:** Overall Survival.

Survival			95% Confidence Interval	
	Estimate	Std. Error	Lower Bound	Upper Bound
Mean	44.99	4.42	36.33	53.65
Median	56.00	N/A	30.00	N/A

## Data Availability

Data will be made available upon request.
